# Tripchlorolide induces cell death in lung cancer cells by autophagy

**DOI:** 10.3892/ijo.2011.1278

**Published:** 2011-12-01

**Authors:** LIMIN CHEN, QING LIU, ZHENGHUI HUANG, FENG WU, ZHIYING LI, XIANGQI CHEN, TINGYAN LIN

**Affiliations:** Department of Respiratory Medicine, Union Hospital, Fujian Medical University, Fuzhou, Fujian 350001, P.R. China

**Keywords:** tripchlorolide, autophagy, A549, lung cancer, apoptosis, LC3

## Abstract

It has been demonstrated that triptolide inhibits the growth of several types of cancer cells *in vitro* and prevents tumor growth *in vivo* by inducing apoptosis and autophagy. Here we showed that Tripchlorolide (T4) significantly suppressed the proliferation of A549 cells in a dose- and time-dependent manner. This suppressive effect was diminished when cells were pretreated with 3-Methylamphetamine (3-MA). After the cells were treated with T4, the LC3 II protein expression was significantly increased, and autophagosomes were observed by TEM. However, almost no apoptosis was observed in A549 treated with T4. These results suggest that T4 induces A549 cell death predominantly through the activation of the autophagy pathway instead of the apoptosis pathway.

## Introduction

Lung cancer is one of the leading causes of cancer death. The overall 5-year survival rate for lung cancer is less than 15% (1), principally due to the metastasis, radiation resistance and drug-resistance in the advanced stage. The main reason for drug treatment failure is the disturbance of the regulation of cell death (2).

Apoptosis is the primary process of cell death that is under complex molecular control and can be triggered by various signals. It is well-documented that apoptosis deficiency in lung cancer results in the drug-resistance (3). Therefore, it is priority for lung cancer treatment to find an alternative process that induces lung cancer death. Recently, many more scientists became interested in autophagy, a cellular process called type II cell death. Central to the process is the formation of autophagosomes, double-membrane vesicles that can be observed by transmission electron microscope (TEM) (4–6). Already in 1980, it was shown that the autophagy capacity of lung cancer is lower than that of surrounding normal cells (7). Furthermore, nutrient deficiency and high cell density increase autophagy capacity (8). Therefore, the decrease of autophagy capacity may be in favor of cancer survival.

The extract from *Tripterygium wilfordii Hook F*, which has been shown to have strong immune inhibition and anti-inflammation activities, is widely used in China to treat certain autoimmune diseases (such as rheumatoid arthritis and kidney diseases) (9). Tripchlorolide (T4) can be extracted from *Tripterygium wilfordii Hook F*, or synthesized from its precursor triptolide after hydroxyl acylation and subsequently chlorination (10). After structure modification, T4 was shown to have similar drug activities, but low toxicities compared to triptolide (11). In the present study, we examined the role of T4 in regulating proliferation, apoptosis and autophagy in lung cancer A549.

## Materials and methods

### Materials

Materials used in this study include 3-Methylamphetamine (3-MA) and PI was purchased from Sigma, anti-MAP-LC3-II from Santa Cruz, IgG-FITC from LingFei, goat anti-rabbit secondary antibody from KPL, β-actin antibody from NeoMarkers, chemiluminescent substrate from KPL, Alex488-conjugated secondary antibody and Topro-3 from Invitrogen.

### Cell culture

Lung cancer cell line A549 was obtained from Cell Line Bank, Chinese Academy of Sciences, and maintained in F-12K supplemented with 10% FBA and 100 μg/ml penicillin and streptomycin at 37°C and 5% CO_2_. Every two days, cell growth media was replaced with fresh media.

### Drug treatment

A549 cells (around 80% confluence) were treated with/without drugs as below: i) control, cells were not treated with drugs; ii) T4 treatment, cells were exposed to T4 as the final concentrations of 5 nM, 25 nM, 50 nM, 100 nM and 200 nM, respectively; iii) 3-MA pretreatment plus T4 treatment, cells were pretreated with 5 mM (final concentration) 3-MA for 1 h, and followed by T4 treatment with various concentrations as indicated above. Cells were collected for the following experiments after they had been treated with/without drugs for 24 h, 48 h or 72 h. Each experiment was performed in triplicates.

### MTT assay

Cells (5×10^3^/well) seeded in a 96-well plate (3 wells per concentration) were exposed to a series of concentrations of T4. The control was supplemented with the same volume of media. Cells were cultured for 0–72 h. MTT (100 μl) (5 g/l) was added to all the wells and the plate was re-incubated at 37°C for 4 h. To each well 90 μl of 10% acid-isopropanol solution was added. After 12 h, the plates were read using a test wavelength of 550 nm with a reference wavelength of 630 nm. The mean OD values of the experiments after calibration were used for calculating the viability rate and the death rate: The viability rate = A550 _sample group/A550 control group_, the death rate = 1 - the viability rate.

### Immunofluorescence assay (IFA)

A549 cells were washed with 0.01 M PBS, fixed in 4% paraformaldehyde solution for 30 min and washed again with PBS. Then A549 were permeabilized by 0.3% Triton X-100 for 10 min and blocked in TBS (5% BSA) for 60 min. After adding anti-MAP1-LC3-II (diluted 1:100), the slides were incubated in a moist chamber at 4°C overnight and subsequently washed again with PBS. The slides were further incubated with Alex 488-labeled secondary antibodies for 1 h in a moist chamber at room temperature and washed again. Topro-3 (diluted 1:100,000 in PBS) was added for nuclear staining and incubated for 10 min, the slides were washed with PBS, and anti-fade mounting medium was added, then covered with cover slip, sealed with nail polish, and examined by fluorescence microscopy. Micrographs were taken for result assessment. The experiment design included negative control and blank control.

### The examination of autophagosome by TEM

Cells were fixed in 3% glutaradehyde/1.5% paraformaldehyde for days or hours at 4°C, and postfixed with 1% OsO_4_/1.5% potassiumferrocyanide for 1.5 h. After washing with PBS, cells were stained with 70% ethanol saturated with uranyl acetate, followed by gradient dehydration with ethanol-acetone, and finally embedded in epoxy resin 618 for section. The ultrathin sections (50 nm) were stained by uranyl acetate and lead citrate for 5 min, respectively, and examined and photographed with TEM.

### Western blotting

Cells were lysed by lysate buffer (10 mmol/l Tris, pH 7.4, 100 mmol/l NaCl, 1 mmol/l EDTA, 1 mmol/l EGTA, 1 mmol/l NaF, 20 mmol/l Na_4_P_2_O_7_, 2 mmol/l Na_3_VO_4_, 0.1% SDS, 0.5% sodium deoxycholate, 1% Triton-X 100, 1 mmol/l PMSF, 60 μg/ml aprotinin, 10 μg/ml leupeptin, 1 μg/ml pepstatin) on ice for 30 min, centrifuged at 12,000 g for 30 min, and collected the supernatant. The supernatant was diluted to 2 mg/ml (protein concentration), mixed 1:1 with loading buffer (62.5 mM Tris-HCl, pH 6.8, 10% glycerol, 2% SDS, and 0.1% bromophenol blue), and boiled for 5 min. After cooling down, samples (30 μg/lane) were loaded and run on a SDS-PAGE (10%) gel. The separated proteins were transferred to PVDF membrane. Membrane was blocked in TBS supplemented with 5% non-fat milk and 0.2% Tween-20 for 1 h, and incubated with primary antibody (diluted 1:500) at 4°C overnight. Following incubation with secondary antibody (coat anti-rabbit HRPO, diluted 1:4,000) for 2 h the membrane was exposed to film. The images of bands were assessed by software from Scion Co. β-actin was used as loading control.

### Statistical analysis

Data are expressed as the mean ± SD (standard deviation) of triplicates. The significance of the differences among the treatments was assessed by one-way analysis of variance (ANOVA). All analyses were conducted with SPSS statistical package. P<0.05 was considered statistically significant.

## Results

### The toxicity of T4

**Figure 1:**
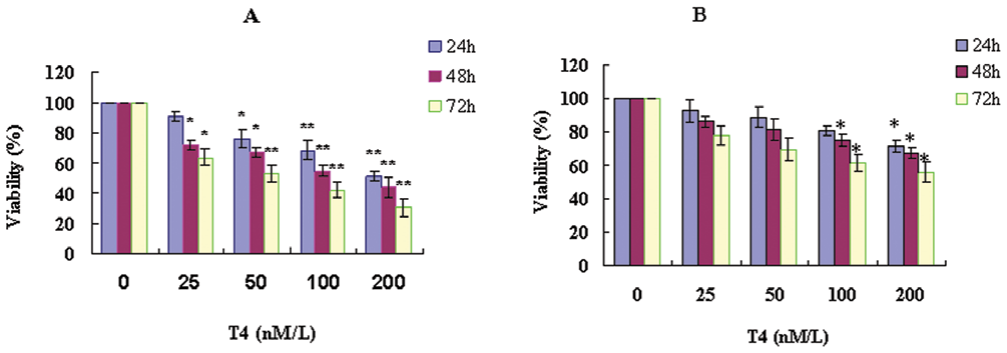
The toxicity of T4. (A) T4 treatment and (B) 3-MA pretreatment followed by T4. (^*^P<0.05, ^**^P<0.01).

A549 cells were treated with a series of concentrations of T4 (0–200 nM) for different periods (24–72 h). The viability of A549 was reduced with increasing T4 concentration and longer incubation time (Fig. 1). After exposure to 200 nM T4 for 24 h, the cells viability declined to around 50%. These results suggested that T4 suppressed the proliferation of A549 in a dose- and time-dependent manner. When the cells were pretreated with 3-MA, the inhibitor of autophagy, T4 toxicity was dramatically reduced. Compared to the controls, the 3-MA pretreated cells followed by T4 treatment were not shown to have significance difference of the cell viability, except those exposed to 100 nM T4 for 48–72 h or 200 nM T4 for 24–72 h.

### T4 rapidly induced A549 autophagy

**Figure 2:**
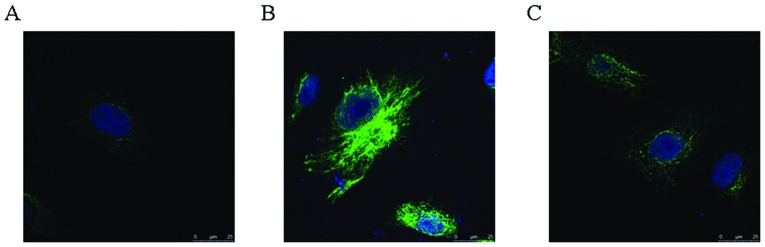
T4 induced A549 autophagy. (A) control. (B) T4 treatment, positive bodies labeled by LC3 increased dramatically (200 nM T4 treatment for 24 h). (C) 3-MA +T4 treatment, LC3 level was decreased compared to the level in the control cells.

Because the IC_50_ for T4 is 200 nM and longer incubation periods dramatically increase the number of dead cells, we chose 200 nM T4 for 24 h treatment as the sample-conditions of IFA. In the control, the results of IFA showed that LC3 (microtubule-associated protein 1 light chain 3) was expressed at low level and distributed in the cytoplasm (Fig. 2). In cells treated with 200 nM T4 for 24 h, the protein expression level of LC3 was dramatically upregulated, and intense granular staining of LC3 was observed. However, in the cells pretreated with 3-MA, LC3 protein level was similar to that in the control. It indicated that T4 can induce A549 autophagy.

### The effect of T4 on apoptosis

**Figure 3:**
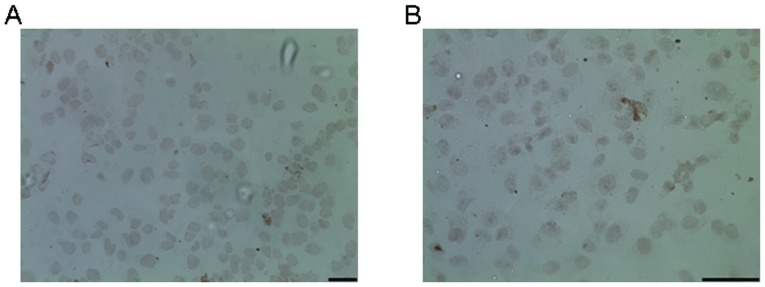
The effect of T4 on A549 apoptosis. (A) Control. (B) Treatment: no obvious positive apoptotic bodies were observed in the cells treated by T4.

To determine whether T4 induced A549 apoptosis or not, we examined the apoptotic bodies in the cells exposed to 200 nM T4 for 24 h. In the treated cells, no typical apoptotic bodies were observed, as revealed by the TUNEL assay (Fig. 3). Therefore, A549 death induced by T4 was not through apoptosis pathway.

### T4 induced autophagosome formation in A549

**Figure 4:**
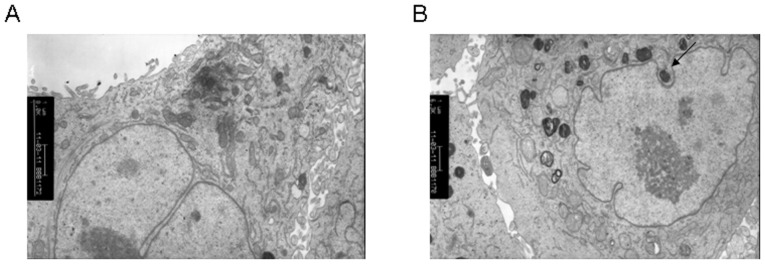
The formation of autophagosomes in A549. (A) control. (B) T4 treated cells: autophagosomes were observed.

To further confirm that T4 can induce A549 autophagy, we checked the formation of autophagosomes in T4 treated cells using TEM. In the control, many normal vesicles containing subcellular organelles were observed (Fig. 4). In the T4 treated cells, damaged organelles were observed, such as swollen mitochondria surrrounded by double-membrane vacuoles, which further formed autophagosomes. Autophagosome subsequently fused with a lysozome and the internal material was degraded. Undegraded debris within the autolysosomes was also observed.

### T4 stimulated LC3 expression in A549 cancer cell

**Figure 5:**
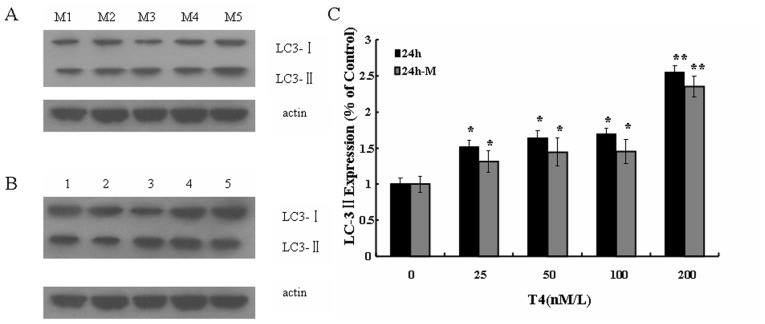
T4 upregulated LC3 expression in A549. 1–5: T4 concentration: 0, 25 nM, 50 nM, 100 nM and 200 nM, respectively. M1-M5: cells were pretreated with 3-MA, T4 concentrations: 0, 25 nM, 50 nM, 100 nM and 200 nM, respectively. (A) T4 treatment; (B) pretreated with 3-MA and followed by T4; (C) T4 treatment significantly upregulated the level of LC3-II in A549.

LC3 is proposed to serve as a marker of autophagosome membrane (16). As determined by Western blotting, the protein level of LC3 II was upregulated with the increase of T4 concentration, and reached the peak at 200 nM T4 treatment (Fig. 5). However, in the 3-MA pretreated cells followed by T4 treatment, the level of LC3 II was much lower than that in the cells only treated by T4, though it was slightly higher than that in the control. These results suggested that T4 stimulated LC3 II expression of A549, while 3-MA weakened the effect. Thus, it was further confirmed that T4 induced cell death in A549 by autophagy.

## Discussion

Autophagy, the type II cell death, is different from apoptosis (type I cell death). Autophagy is a strictly controlled process in regulating cellular contents degradation and recycle, and plays an essential role in organelles metabolism and bio-energy supplementation. Malfunction of autophagy has been associated with many human diseases, such as cancer and neurodegene-rative disorders (12). Cancer is one of the earliest diseases found to be related to autophagy (13). Autophagy inhibits tumorigenesis mainly through regulation of the concentration of cellular peroxide, diminishing the disturbance of protein metabolism and maintenance of cell homeostasis. The decrease of autophagy activity promotes oxidation stress and thereby increases accumulation of cancer-related mutants (14).

Autophagic activity of cancer cells is lower than that of normal cells (7). The decrease of autophagic capacity is also found in precancerous cells of some cancers, such as hepatocarcinoma induced by chemical carcinogen. Reduced autophagic capacity is in favor of carcinomagenesis. It is necessary for cells to maintain a normal acutophgic capacity for cleaning the damaged organelles due to chemical carcinogens, radioation and oxidation stress, and thus maintaining genome integrity and reducing tumor occurrence. Secondly, autophagy degrades organelles (such as endoplasmic reticulum and Golgi) and long-lived protein, and thereof results in negative protein balance in premalignant cells, inhibiting uncontrolled proliferation (15). In addition, hyperactive autophagy induces tumor cell death (16–18).

Recently, it has been reported that some herbal drugs were used for cancer treatment due to their effect on cell autophagy. For exempt, Aloe-emodin, a herbal anthraquinone derivative, induced rat C6 glioma autophagic death (19). Reseratrol, a natural phytoalexin present in grapes, nuts and red wine, induced ovarian cancer cell death through autophagy (20). 6-Shogaol, an active component from ginger, induced A549 autophagy by suppressing the AKT/mTOR pathway (21). Triptolide, the precursor of T4, inhibited the growth of hamster cholangiocarcinoma (22) and human tumors transplanted into nude mice (23). Several studies revealed that triptolide suppressed the growth of pancreatic cancer and neuroblastoma both *in vivo* and *in vitro* (24,25). Nammeeta *et al* found that triptolide induced pancreatic cancer cell death through apoptosis and autophagy (26). Furthermore, triptolide in combination with TRAIL significantly decreased pancreatic cancer cell viability (27). However, the role of triptolide in regulating lung cancer cells has not been reported. The clinical application of the crude extract from *Tripterygium wilfordii* is restricted due to its high toxicity.

In the present study, we studied the role of T4 in A549 lung cancer cells. MTT results showed that T4 induced cytoxity in A549 cell death in a dose- and time-dependent manner. Autophagosomes were observed by TEM in A549 treated by T4. When A549 was pretreated with 3-MA and followed by T4 treatment, the mortality of A549 was much lower than that in A549 only exposed to T4. Although apoptosis adduced by triptolide was found in various cancer cells (26,28–30), almost no apoptosis was observed in A549 treated T4. Therefore, T4 suppressed the proliferation of A549 principally through activating autophagy pathway, but not apoptosis pathway. A recent study showed that triptolide promotes lung cancer apoptosis dependent on TRAIL (31). Further studies are required to investigate the effect of T4 in combination with TRAIL on lung cancer cells.

Autophagosome formation is known to have a central role in autophagy. Two ubiquitin-like conjugation systems are essential for the formation. One is Atg12-Atg5 conjugation system, and the other is Atg8 regulated lipidation system (15). LC3, the mammalian homologue of Atg8, has three subtypes: pre-LC3, LC3-I and LC3-II. pre-LC3 is processed by the cleavage of a portion (22 amino acids) of its C-terminal to form LC3-I, which is distributed in the cytoplasm. LC3-I is converted to LC3-II by further cleavage and conjugation to phosphatidylethanolamine. LC3-II is recruited on the membrane of pre-autophagosome by Atg5 and still located on autophagosome membrane after autophagosome formation. Therefore, LC3-II is proposed to serve as a marker of cell autophagy (15). IFA results showed that the number of LC3-positive granules was significantly increased in A549 treated by T4. However, in the 3-MA pretreated cells, followed by T4 treatment, autophagosomes were dramatically reduced compared to the control. Moreover, the protein level of LC3-II was upregulated after T4 treatment in a time- and concentration-dependent manner, and reached the peak at 200 nM. 3-MA pretreatment dramatically reduced the protein level of LC3-II in A549, though it was slightly higher than that of the control. These results indicate that T4 kills lung cancer cells by promoting autophagy, which is different from the mechanism of anti-cancer effect of triptolide for other cancers (26,28–30).

In conclusion, our study confirmed that T4 suppressed the proliferation of A549 cells by activating autophagy. The study provides new evidence for T4 clinical application in lung cancer treatment.
